# Progress in immunotherapy for brain metastatic melanoma

**DOI:** 10.3389/fonc.2024.1485532

**Published:** 2025-01-28

**Authors:** Shicheng Zheng, Zhongqiao Lin, Ruibo Zhang, Zihang Cheng, Kaixin Li, Chenkai Gu, Yu Chen, Jing Lin

**Affiliations:** ^1^ School of Basic Medicine, Fujian Medical University, Fuzhou, Fujian, China; ^2^ Phase I Clinical Trial Ward, Clinical Oncology School of Fujian Medical University, Fujian Cancer Hospital, Fuzhou, Fujian, China; ^3^ Department of Medical Oncology, Clinical Oncology School of Fujian Medical University, Fujian Cancer Hospital, Fuzhou, Fujian, China; ^4^ Cancer Bio-Immunotherapy Center, Clinical Oncology School of Fujian Medical University, Fujian Cancer Hospital, Fuzhou, Fujian, China

**Keywords:** melanoma, brain metastasis, immunotherapy, immune checkpoint inhibitors, combination therapy

## Abstract

Melanoma is highly aggressive, with brain metastasis being a significant contributor to poor outcomes. Immunotherapy has emerged as a crucial treatment modality for melanoma, particularly for addressing brain metastases. This review explores recent developments in immunotherapy for patients with melanoma brain metastasis, with such treatments encompassing immune checkpoint inhibitors and various immunotherapy combination approaches, such as dual immunotherapy, immunotherapy combined with chemotherapy, immunotherapy combined with targeted drugs, and immunotherapy combined with radiotherapy. This article also discusses existing treatment obstacles and potential future avenues for research and clinical practice.

## 1 Introduction

A melanoma is a malignant tumor arising from melanocytes located in the skin, mucosa and other tissues. The disease is categorized into cutaneous, mucosal, arcal, and unknown primary types on the basis of the site of origin (1, 2). Melanoma exhibits a high degree of malignancy and aggressiveness and is susceptible to distant metastasis. Indeed, patients with melanoma have a greater prevalence of brain metastases than patients with other cancers (3), with an incidence of 28%-60% at diagnosis or during treatment and 73%~90% in postmortem (4–6). Melanoma brain metastases (MBMs) also have a poor prognosis in the past, with dismal patient survival durations of 3-6 months (4, 7–9). Early treatment of MBMs is particularly challenging because many therapeutic agents cannot penetrate the blood−brain barrier (BBB) to reach the brain (10). Chemotherapy, which commonly involves temozolomide and fotrmustine, has an intracranial objective response rate (ORR) of only 10%-14.3% (11, 12). Radiation therapy is typically indicated for patients presenting with meningeal or diffuse brain metastases; these patients have a median survival generally limited to approximately three months following localized interventions, including surgery and radiation therapy (10). In addition, patients with MBMs have often been excluded from clinical studies (13). Currently, the development of immunotherapies, such as immune checkpoint inhibitors, is crucial for the treatment of brain metastatic melanoma. Novel treatment approaches such as immunotherapy and combination immunotherapy have enabled clinicians to adopt more targeted and strategic approaches for the treatment of these patients. This article reviews the recent progress in immunotherapy and its combination with chemotherapy, radiotherapy, targeted drugs, as well as other immune therapeutic modalities in the treatment of MBMs.

## 2 Progress in immune checkpoint inhibitors and cytologic immunotherapy

### 2.1 Progress in immune checkpoint inhibitor monotherapy

Currently, immune checkpoint inhibitors such as CTLA-4, PD-1/PD-L1, LAG-3 monoclonal antibodies and TIGIT monoclonal antibodies are widely used for immunotherapy or during clinical trials and show desirable efficacy (14, 15). For melanoma patients with brain metastases, the common immune checkpoint inhibitors used for monotherapy are PD-1/PD-L1 and cytotoxic T lymphocyte-associated protein 4 (CTLA-4). CTLA-4 inhibitors, such as ipilimumab, can enhance and prolong the adaptive immune response to tumor cells by blocking the CTLA-4 molecule. In addition, ipilimumab is the first commercialized immune checkpoint inhibitor. A multicenter phase II study of ipilimumab in 2012 was the first to report the intracranial activity of an immune checkpoint inhibitor in patients with MBMs (16). However, only patients with asymptomatic brain metastases show greater benefit, with response rates of 16% and a median overall survival of 7.0 months (16). Therefore, the following studies of ipilimumab have focused more on combinations with PD-1 inhibitors (e.g., Checkmate 204). Other CTLA-4 inhibitors, such as tremelimumab, are currently unreported for the treatment of MBMs.

PD-1 (Programmed cell death protein 1) and PD-L1 (Programmed death-ligand 1) inhibitors can reactivate the immune response of T cells to tumors by blocking the binding of PD-1 to PD-L1 (14, 17). In MBMs, PD-1/PD-L1 inhibitors such as pembrolizumab and nivolumab have demonstrated marked therapeutic efficacy (18). A phase II study initially demonstrated a response rate of 22% in 18 melanoma patients with asymptomatic brain metastases measuring <2 cm who received pembrolizumab, and their durations of brain metastasis response were all greater than 4 months (19). Patients with asymptomatic brain metastases benefitted more from nivolumab, with response rates of 20% and 18.5 months of median overall survival. This multicohort study revealed that patients with symptomatic brain metastases had a response rate of only 6% and a median overall survival of 5.1 months, regardless of the presence of BRAF mutations (20). Another retrospective study revealed that patients with symptomatic brain metastases had shorter PFS than did those without symptoms (2.7 vs. 7.4 months, P=0.035), as well as a lower ORR (21% vs. 56%), regardless of which anti-PD-1 therapy (pembrolizumab or nivolumab) was used (21). Thus, anti-PD-1 monotherapy may be suitable for patients with asymptomatic brain metastases. PD-L1 inhibitors such as atezolizumab, durvalumab, and avelumab have not been studied or reported to be effective for monotherapy in MBMs thus far.

### 2.2 Progress in ICI dual-immunotherapy

ICI monotherapy has been shown to be effective in patients with symptomatic brain metastases. Several studies have demonstrated that the combination of ICIs yields a higher intracranial remission rate and greater efficacy than individual ICIs do (22). Nivolumab and ipilimumab stand out as the most frequently utilized combination among the various treatment options (23). The ABC study first assessed the effectiveness of nivolumab (anti-PD-1) combined with ipilimumab (anti-CTLA-4) in the treatment of MBMs. In the recent 5-year follow-up, the intracranial response (ICR) rates were 51% in patients in cohort A (nivolumab + ibritumomab, asymptomatic), 20% in cohort B (nivolumab, asymptomatic), and 6% in Cohort C (nivolumab; patients who failed local therapy or experienced neurologic symptoms). The 5-year intracranial PFS rates were 46% in cohort A, 15% in cohort B, and 6% in cohort C (24). Further research, such as the CheckMate 204 study, included long-term evaluations of the efficacy of nivolumab plus ipilimumab. The study revealed that 54% (54/101) of asymptomatic patients experienced clinical remission, and 33% of them achieved an intracranial complete response; the 36-month intracranial progression-free survival rate was 54.1%, and the overall survival rate was 71.9%. In contrast, among symptomatic patients, only 16.7% (3/18) had an intracranial complete response; the 36-month intracranial progression-free survival rate was 18.9%, and the overall survival rate was 36.6%. In addition, only 15% of patients in the CheckMate 204 study experienced grade 3–4 treatment-related adverse events (TRAEs), which were well tolerated (25). Compared with that of previous chemotherapy, which has achieved a 10%-14.3% remission rate for brain metastases (11, 12), the efficacy of ICI dual-immunotherapy has significantly improved. In the CheckMate 204 study, the NCCN, ESMO, EORTC, and CCA guidelines recommended nivolumab plus ipilimumab as the preferred initial treatment in untreated asymptomatic patients with MBM < 3 cm (26). Another retrospective study included patients with symptomatic MBMs and concurrent treatment with corticosteroids who were also receiving ipilimumab plus nivolumab. The researchers reported an objective response rate (ORR) of 28% (8/29) and a duration of response (DOR) of 7.85 months; however, the responding patients had a longer OS of 56.4 months (27). Patients with symptomatic MBMs only modestly benefit from ICI dual immunotherapy, possibly because steroids impair the efficacy of ICIs, which still need more evidences. Regarding other ICI combinations, a recent phase II study of relatlimab (LAG-3 monoclonal antibody) used in combination with nivolumab in patients with active MBMs is in progress (NCT05704647), and the results are keenly anticipated (28). **Table 1** summarizes the ORR, intracranial PFS and OS of the mono-ICI and dual-ICI trials mentioned above.

**Table 1:** Outcomes of patients with melanoma brain metastases treated with ICIs.

First author (Year)	Study design	Number of patients	BRAF mutated	Intervention	ORR	Intracranial PFS	OS
Margolin K (2012) (16)	Open-label phase II	72 (51 asymptomatic patients and 21 symptomatic patients)	Not mentioned	Four doses of intravenous ipilimumab 10 mg/kg, Q3W	16% in asymptomatic patients 5% in symptomatic patients	2.7 months in asymptomatic patients 1.3 months in symptomatic patients	7.0 months in asymptomatic patients 3.7 months in symptomatic patients
Kluger H M (2019) (18)	Phase II	23 (asymptomatic or symptomatic not mentioned)	41%	Pembrolizumab 10 mg/kg, Q2W for 2-year	35%	2 months	17 months
Goldberg S B (2016) (19)	Nonrandomized open-label phase II	18 with MBM (asymptomatic or symptomatic not mentioned)	33%	Pembrolizumab 10 mg/kg, Q2W	22%	Not mentioned	Not mentioned
LONG G V (2018、2021) (20, 24)	Multicohort phase II	79 (36 asymptomatic patients in cohort A, 27 asymptomatic patients in cohort B and 16 symptomatic patients in cohort C)	81%	Nivolumab 1 mg/kg + ipilimumab 3 mg/kg, Q3W for four doses, then nivolumab 3 mg/kg, Q2W (Corhort A) Nivolumab 3 mg/kg, Q3W (Corhort B & C)	46% in asymptomatic patients for cohort A 20% in asymptomatic patients for cohort B 6% in symptomatic patients for cohort C	46% of 5-year rate in asymptomatic patients for cohort A 15% of 5-year rate in asymptomatic patients for cohort B 6% of 5-year rate in symptomatic patients for cohort C	Not reached in asymptomatic patients for cohort A 18.5 months in asymptomatic patients for cohort B 5.1 months in symptomatic patients for cohort C
Parakh S (2017) (21)	Retrospective study	66 (46 asymptomatic patients and 20 symptomatic patients)	45%	Pembrolizumab or nivolumab, with dosages not mentioned	56% in asymptomatic patients 21% in symptomatic patients	7.4 months in asymptomatic patients 2.7 months in symptomatic patients	13.0 months in asymptomatic patients 5.7 months in symptomatic patients
Tawbi H A (2021) (25)	Multicohort phase II	119 (101 asymptomatic patients and 64 symptomatic patients)	62%	Nivolumab 1 mg/kg + ipilimumab 3 mg/kg, Q3W for four doses, then nivolumab 3 mg/kg, Q2W	53.5% in asymptomatic patients 16.7% in symptomatic patients	39.3 months in asymptomatic patients 1.2 months in symptomatic patients	71.9% of 3-year rate in asymptomatic patients 36.6% of 3-year rate in symptomatic patients
Manacorda S (2023) (27)	Retrospective study	29 (all symptomatic patients)	48%	Nivolumab + ipilimumab, dosages not mentioned	28%	Not mentioned	5.45 months

### 2.3 Progress in cytologic immunotherapy

In addition to well-known immune checkpoint inhibitors, oncolytic virus immunotherapy, tumor-infiltrating lymphocyte (TIL) therapy and TCR-T-cell therapy are novel individualized immunotherapies. The oncolytic virus T-VEC has shown good efficacy in the treatment of extracranial lesions and has been approved by the FDA for clinical treatment. It is also considered useful in brain metastasis patients because it is capable of crossing the BBB, but few data on its use combined with ICIs in clinical trials are available at present. There are only two clinical cases showing that T-VEC has a certain effect on brain metastatic lesions, and its specific efficacy still needs to be further explored in trials (29, 30). TIL therapy attacks tumor cells by extracting and modifying T cells from the patient’s own tumor tissue and then reinjecting them into the patient’s body. In a clinical trial, Lifileucel, a novel TIL therapy, was used to treat refractory melanoma. The study, which included 153 patients, including those with brain metastases, demonstrated an objective remission rate (ORR) of 31.4%, a median survival (OS) of 13.9 months, and a 12-month OS incidence of 54.0% (31). Lifileucel initially showed good antitumor activity in patients with advanced brain metastatic melanoma and was also effective in patients who had failed PD-1 therapy, with a long-lasting clinical benefit as well as a high ORR (31). T-cell receptor–engineered T (TCR-T) cell therapy has several advantages, such as a large repertoire of targetable antigens, lower epitope density, increased sensitivity and greater avidity, which have revolutionized the immunotherapy of cancers (32). However, TCR-T-cell therapy for MBMs is still in its infancy. There is only one case report, which describes a mucosal melanoma patient with brain metastases who received MAGE-A4-targeted TCR-T-cell therapy in combination with low-dose radiotherapy and experienced durable remission (33). Cytologic therapy has initially shown notable efficacy in the treatment of MBM, but its effectiveness and safety need to be fully explored.

## 3 Progress in immunotherapy combined with chemotherapy

Chemotherapy, such as temozolomide or fotemustine, is less effective in controlling MBM, with an intracranial ORR of 10%-14.3% (11, 12). Chemotherapy combined with immunotherapy for MBM is less studied. Fotemustine is a chemotherapeutic drug commonly used for patients with MBM, and the overall remission rate was found to be 17% in patients receiving monotherapy (12). A multicenter phase III NIBIT-M2 trial explored the efficacy of the combination of fotemustine with ipilimumab in MBM. The last 7-year follow-up study reported a 7-year intracranial ORR of 19.2% for fotemustine plus ipilimumab, 11.5% for the 7-year intracranial PFS rate and 13.8 months for the median intracranial DOR (34). Although the NIBIT-M2 trial did not demonstrate favorable outcomes of ORR and PFS with ipilimumab + fotemustine compared with ipilimumab + nivolumab, Grade 3~4 immune-related AEs were observed in 38% (10) of patients receiving ipilimumab plus fotemustine (34), similar to the 33% (9) receiving ipilimumab + nivolumab. Unlike the NIBIT-M2 trial (anti-CTLA-4 combination with chemotherapy), anti-PD-1/PD-L1 combination with chemotherapy has been less explored in MBM but has shown efficacy in advanced nonsquamous NSCLC patients with brain metastases, with a confirmed intracranial ORR of 46.7% and an intracranial PFS of 7.6 months (CAP-BRAIN trial) (35). Future studies may focus on this combination and explore the efficacy of MBM.

In acral melanoma with brain metastases, heterogeneous tumor interactions within the brain microenvironment drive resistance to ICIs and target drugs (36). Under such circumstances, chemotherapy may remain important as standard therapy. In a recent CAP-03 trial, camrelizumab + apatinib + temozolomide triple therapy showed primary efficacy in advanced acral melanoma without brain metastases (37). The ORR was 64.0%, the DOR was 17.5 months, and the median PFS was 18.4 months (37). Although grade 3 or 4 treatment-related AEs (TRAEs) have been reported in 66% of patients, they can be effectively alleviated by dosage adjustments and symptomatic treatments (37). Although the CAP-03 trial did not include patients with brain metastases, this triplet combination may provide new insights into the treatment of acral melanoma with brain metastases.

Furthermore, for patients with symptomatic MBM, who may receive steroids to temporarily alleviate symptoms of increased intracranial pressure and cerebral edema, immunotherapy combined with chemotherapy may be advantageous, as chemotherapy can help control cerebral lesions before the addition of immunotherapy for a synergistic effect. However, no retrospective or prospective studies on immunotherapy combined with chemotherapy in patients with symptomatic MBM have been conducted, and further research in such patients is needed.

## 4 Progress in immunotherapy combined with targeted drugs

The classical COMBI-MB study demonstrated promising treatment efficacy of dabrafenib + trametinib (D+T) in patients with BRAF V600-mutated MBM, with an intracranial ORR of up to 58% and a median PFS of up to 5.6 months (38). However, evidence for the combination of immunotherapy and targeted therapy for MBM patients with BRAF mutations is lacking. Although of three large randomized controlled studies, KEYNOTE-022, IMspire150, and COMBI-I for the triplet regimen of immunotherapy combined with targeted drugs in advanced melanoma patients with BRAF V600 mutations, none demonstrated meaningful improvements in OS, and all three trials reported increases in adverse events in the triple therapy group (39–41). However, IMspire150 likely presented a longer median PFS (15.1 vs. 10.6 months) and median DOR (21.0 vs. 12.6 months) and a 23% reduction in disease progression and a 21% reduction in death risk in the triple therapy group than in the dual therapy group (42). A retrospective analysis revealed that patients who undergo at least six months of targeted therapy without disease progression tend to experience better remission rates when subsequent immunotherapy is administered than do those who experience rapid relapse (43). For MBM patients, an important study of immunotherapy combined with targeted drugs was performed in the TRICOTEL trial, which showed that the addition of atezolizumab (anti-PD-L1) to vemurafenib (BRAFi) or cobimetinib (MEKi) provided promising intracranial activity in patients with BRAF V600-mutated melanoma with CNS metastases, the intracranial ORR was 42% in the BRAF V600 mutation-positive cohort and 27% in the BRAF V600 wild-type cohort (44). In patients with BRAF V600 mutations, the intracranial ORR was 35% in symptomatic patients, which was lower than the 46% reported in asymptomatic patients. The intracranial DOR was 7.4 months and the PFS was 4.5 months in symptomatic patients, whereas the intracranial DOR was 7.6 months and the PFS was 5.5 months in asymptomatic patients, but the difference was not statistically significant (44). Treatment-related serious adverse events (grade ≥3) occurred in 16 (21%) of 75 patients who received triplet therapy, and no treatment-related deaths occurred, which initially indicated favorable safety (44). The ongoing phase II SWOG S2000 trial is aimed to explore a triplet regimen of BRAF/MEK inhibitors with anti-PD-1 therapy (encorafenib + binimetinib + nivolumab) versus anti-PD-1+CTLA-4 therapy (nivolumab + ipilimumab) in patients with symptomatic BRAF-mutant MBM; these results are also highly anticipated (45).

## 5 Progress in immunotherapy combined with radiotherapy

Radiotherapy (RT) is a crucial local treatment for tumor brain metastases, and common radiation strategies include whole-brain radiotherapy (WBRT) and stereotactic radiosurgery (SRS) (46). Whole-brain radiation therapy (WBRT) offers a robust solution for managing both multiple and solitary lesions and is used in patients with multiple MBMs but has a limited effect on median survival (26). Stereotactic radiosurgery (SRS) delivers a high dose of radiation and can focus on certain areas with high three-dimensional conformality, which is effective in controlling a small number (< 4) of MBM lesions (with a total cerebral tumor volume of < 5 cm^3^) (26). Radiotherapy has been proven to improve immunosuppression in the tumor microenvironment, increase the permeability of the blood–brain barrier, promote T-cell activation by stimulating IFN-γ production and increasing MHC Class I, promote tumor antigen presentation, upregulate PD-L1 expression on the surface of tumor cells, and increase the efficacy of immune checkpoint inhibitors (47–51). Currently, RT combined with ICI therapy holds tremendous promise for controlling MBM. Potential sympathetic effects of immunotherapy combined with radiotherapy in the treatment of MBMs have been reported in several studies. A large meta-analysis including 44 studies indicated better survival outcomes in the ICI + RT group than in the RT alone or ICI alone groups (52). In addition, there were no marked increases in Grade 3~4 neurologic adverse events (NAEs) or Grade ≥ 3 radiation necrosis (52) in the ICI + RT group. A real-world study revealed that WBRT combined with ICIs significantly increased the median overall survival to 4.89 months compared with 3.12 months for WBRT alone, but Cox regression revealed that WBRT + ICI was associated with an increased risk of death (53). As WBRT poses a risk of cognitive impairment, three studies comparing SRS to WBRT in patients with 1-3 brain metastases revealed that SRS not only mitigated the detrimental effects of radiotherapy on cognitive function but also increased patient survival rates (54–56). A retrospective analysis included 160 patients with MBMs treated with SRS in combination with nivolumab, which demonstrated local control rates of 91% and 85% at 6 months and 12 months, respectively, along with OS rates of 11.8 and 12.0 months, respectively, which were significantly greater than those of patients treated with nivolumab or SRS alone (57). No treatment-related neurologic toxicities (such as nausea, vision changes, or focal weakness) or scalp reactions were reported during or after radiation (57).

For asymptomatic patients with MBMs, the dose−size response relationship of SRS with ICIs showed that the 12-month local control rate for a 7.5 mm lesion subjected to SRS (18 Gy) with ICIs was 87.8%, which was noticeably higher than the 79.8% reported without ICIs (58). Further studies compared the efficacy of SRS + nivolumab and SRS + ipilimumab in patients with either asymptomatic or symptomatic MBMs. SRS + nivolumab resulted in meaningful intracranial remission, resulting in 6-month and 12-month intracranial PFS rates of 69% and 42%, respectively, compared with 48% and 17%, respectively, for SRS + ipilimumab (59). Furthermore, the extracranial PFS and OS rates were 37% and 78% in the SRS + nivolumab group, respectively (59). Another notable finding in this study was that patients receiving multifraction SRS (3×9 Gy) compared with those receiving single-fraction SRS had better intracranial PFS (70% versus 46% at 6 months, p = 0.01), especially in combination with nivolumab (59). Moreover, Grade 3 AEs (e.g., diarrhea and fatigue) occurred in 11% of SRS + ipilimumab patients and in 6% of SRS + nivolumab patients, and radiation-induced brain necrosis occurred in 15% of all patients (59). SRS combined with dual-ICI immunotherapy was also explored. One study evaluated the clinical outcomes of patients with MBM treated with SRS within 3 months of receiving anti-PD-1+CTLA-4 therapy, anti-PD-1 therapy, or anti-CTLA-4 therapy; however, the 12-month OS rate and PFS rate for patients receiving SRS + anti-PD-1 + CTLA-4 therapy (68%, 57%) were higher than those for patients receiving SRS + anti-PD-1 therapy (59%, 53%) and SRS + anti-CTLA-4 therapy (45%, 42%), and the local control rates did not differ between the groups, with adverse effects on the rates of radiation necrosis (lower than 7%) (60). However, grade 1–2 toxicity was noted to be highest in patients treated with SRS+anti-PD-1+CTLA-4 therapy (45%) (60). Treatment with ICIs combined with SRS enhances the control of MBM and prolongs OS, and anti-PD-1 therapy combined with SRS is more recommended.

However, whether concurrent radiotherapy with ICIs or subsequent radiotherapy with ICIs is more effective and safer needs to be considered. Concurrent therapy was predominantly defined as the interval between the administration of an ICI and SRS within 4 weeks, and subsequent therapy was defined as the initiation of ICI treatment either >4 weeks before or after SRS (61). An international meta-analysis including 17 individual studies demonstrated that concurrent therapy yielded a higher 1-year OS rate (64.6%) than did subsequent therapy (51.6%), but the local control rate at 1 year did not significantly differ (89.2% vs. 67.8%, p = 0.09) (62). Thus, concurrent therapy may lead to better long-term outcomes. However, in the context of concurrent therapy, whether it is more appropriate to start with RT or ICI therapy still unclear. An exploratory phase II trial revealed that when MBM patients received RT (SRS or WBRT depending on the number of MBMs) followed by ipilimumab (ipi) ± nivolumab (nivo) at 3 weeks, after RT plus two cycles of ipi-based ICI, increased frequencies of activated CD4+ and CD8+ T cells and an increase in melanoma-specific T-cell responses were observed in the peripheral blood, suggesting that sequencing RT followed by ICI treatment may yield better outcomes in MBM patients (63). Thus, although immunotherapy combined with radiotherapy has been proven effective against MBMs, its treatment dosage and sequence require further study.

## 6 Conclusions

Malignant MBMs have a high incidence and poor prognosis. In the past, neurosurgery and radiotherapy were the primary treatments for patients with brain metastases. Since then, the emergence of immunotherapy has led to new treatment options for melanoma brain metastasis. Drugs targeting PD-1/PD-L1 and CTLA-4 have demonstrated efficacy in treating brain metastases. Newly marketed TIL cell therapies in recent years have also demonstrated notable efficacy, and many novel immunotherapies, such as CDR1as, novel TCR-T cells, and novel oncolytic viruses, have shown greater promise in the treatment of brain metastases. Clinical trials have shown that immunotherapy combined with other treatments can improve the treatment efficacy in MBM patients. However, the adverse reactions caused by combination therapy cannot be ignored, as these adverse reactions have a higher incidence rate and a significantly higher degree and the discontinuation of drug therapy caused by these adverse reactions is extremely unfavorable for the treatment of brain metastases. These advancements underscore the evolving landscape of treatment for MBMs, offering new hope and options for patients.
